# [2,9-Bis(3,5-dimethyl-1*H*-pyrazol-1-yl-κ*N* ^2^)-1,10-phenanthroline-κ^2^ *N*,*N*′](methanol-κ*O*)(nitrito-κ^2^ *O*,*O*′)cadmium(II) perchlorate

**DOI:** 10.1107/S1600536811003813

**Published:** 2011-02-12

**Authors:** Li Zhen Liu, Yan Hui Chi, Hua Du, Jing Min Shi

**Affiliations:** a College of Chemistry, Chemical Engineering and Materials Science, Shandong Normal University, Jinan 250014, People’s Republic of China

## Abstract

In the title complex, [Cd(NO_2_)(C_22_H_20_N_6_)(CH_3_OH)]ClO_4_, the Cd^II^ ion is in a distorted penta­gonal–bipyramidal CdN_4_O_3_ coordination geometry. The dihedral angles formed between the mean planes of the pyrazole rings and the phenanthroline ring system are 4.37 (19) and 5.84 (21)°. In the crystal, the anions and cations are connected by inter­molecular O—H⋯O hydrogen bonding, while pairs of weak inter­molecular C—H⋯O hydrogen bonds connect the cations into centrosymmetric dimers. In addition, there is a π–π stacking inter­action involving two symmetry-related benzene rings, with a centroid–centroid distance of 3.437 (3) Å.

## Related literature

For a related structure, see: Zheng & Chi (2011 ▶).
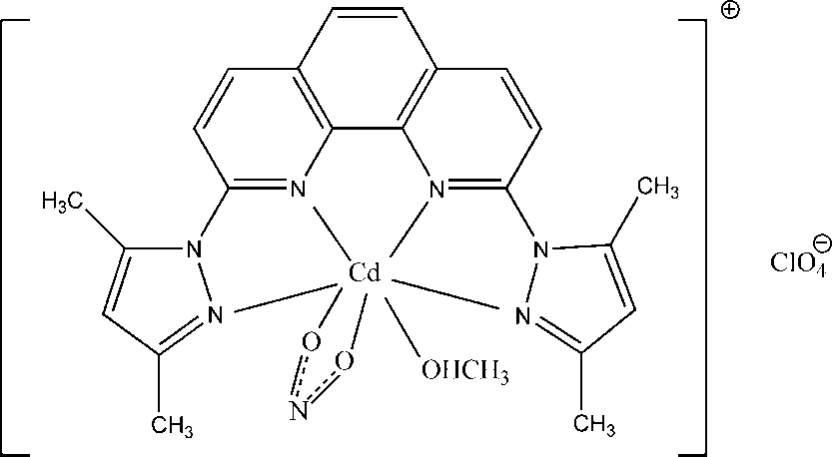


## Experimental

### 

#### Crystal data

[Cd(NO_2_)(C_22_H_20_N_6_)(CH_4_O)]ClO_4_
*M* *_r_* = 658.34
Triclinic, 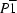

*a* = 8.0241 (17) Å
*b* = 11.580 (2) Å
*c* = 15.842 (3) Å
α = 68.595 (2)°
β = 75.578 (2)°
γ = 73.616 (3)°
*V* = 1297.1 (5) Å^3^
*Z* = 2
Mo *K*α radiation
μ = 1.00 mm^−1^
*T* = 298 K
0.32 × 0.08 × 0.04 mm

#### Data collection

Bruker SMART APEX CCD diffractometer
Absorption correction: multi-scan (*SADABS*; Sheldrick, 1996 ▶) *T*_min_ = 0.740, *T*_max_ = 0.961
6780 measured reflections
4712 independent reflections
3966 reflections with *I* > 2σ(*I*)
*R*_int_ = 0.025

#### Refinement

*R*[*F* ^2^ > 2σ(*F* ^2^)] = 0.044
*wR*(*F* ^2^) = 0.111
*S* = 1.02
4712 reflections
357 parameters
1 restraint
H-atom parameters constrained
Δρ_max_ = 0.88 e Å^−3^
Δρ_min_ = −0.51 e Å^−3^

### 

Data collection: *SMART* (Bruker, 1997 ▶); cell refinement: *SAINT* (Bruker, 1997 ▶); data reduction: *SAINT*; program(s) used to solve structure: *SHELXTL* (Sheldrick, 2008 ▶); program(s) used to refine structure: *SHELXTL*; molecular graphics: *SHELXTL*; software used to prepare material for publication: *SHELXTL*.

## Supplementary Material

Crystal structure: contains datablocks I, global. DOI: 10.1107/S1600536811003813/lh5203sup1.cif

Structure factors: contains datablocks I. DOI: 10.1107/S1600536811003813/lh5203Isup2.hkl

Additional supplementary materials:  crystallographic information; 3D view; checkCIF report

## Figures and Tables

**Table 1:** Hydrogen-bond geometry (Å, °)

*D*—H⋯*A*	*D*—H	H⋯*A*	*D*⋯*A*	*D*—H⋯*A*
O7—H9⋯O2^i^	0.87	2.02	2.892 (7)	172
C8—H8⋯O6^ii^	0.93	2.47	3.291 (6)	148

Symmetry codes: (i) 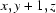; (ii) 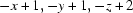.

**Fig. 1.**
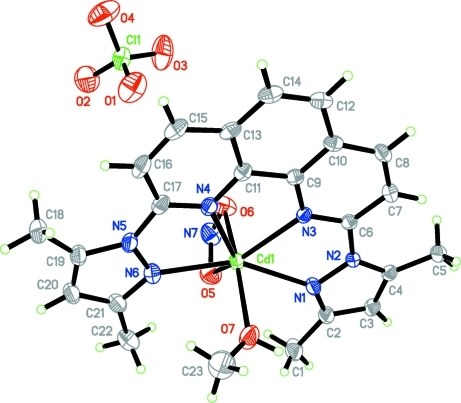
The asymmetric unit of the title compound with displacement ellipsoids shown at the 30% probability level.

## supplementary crystallographic information

### Comment

Derivatives of 1,10-phenanthroline play an important role in modern coordination chemistry and many complexes have been reported with these types of compounds as ligands [see e.g. Zheng & Chi (2011) for a closely related Cd complex]. To the best of our knowledge, the above cited structure is the only other complex reported to date containing a 2,9-bis(3,5-Dimethyl-1H-pyrazol-1-yl)-1,10-phenanthroline ligand. Herein we report the crystal of the title compound (I).

The molecular structure of the title compound is shown in Fig. 1. The Cd^II^ ion is in a distorted pentagonal bipyramidal coordination geometry, which may be attributed to the chelation modes of the 2,9-bis(3,5-Dimethyl-1H-pyrazol-1-yl)-1,10-phenanthroline ligand and nitrite anion ligand. The dihedral angles between the planes that consist of the non-hydrogen atoms of the 1,10-phenanthroline ring system and the pyrazole rings are 4.37 (19)° (involving the pyrazole ring containing atoms N1 and N2) and 5.84 (21)° (involving the pyrazole ring containing atoms N5 and N6), respectively. In the crystal, the anion and cation are connected by an intermolecular O—H···O hydrogen bond, while pairs of weak intermolecular C—H···O hydrogen bonds connect cations into centrosymmetric dimers. In addition, there is a π–π stacking interaction involving symmetry-related complexes, the relevant distance being Cg1···Cg1^i^ 3.437 (3) Å and Cg1···Cg1^i^_perp_ = 3.378 Å (symmetry code: (i) 2-x, 1-y, 2-z; Cg1 is the centroid of the C9-C14 benzene ring; Cg1···Cg1^i^_perp_ is the perpendicular distance from Cg1 ring to Cg1^i^ ring).

### Experimental

A 5 ml H_2_O solution of NaNO_2_ (0.0310 g, 0.449 mmol) was added into 8 ml methanol solution of Cd(ClO_4_).6H_2_O (0.0939 g,0.224 mmol) and the solution was mixed with a 10 ml dichloromethane solution of 2,9-bis(3,5-Dimethyl-1*H*-pyrazol-1-yl)-1,10-phenanthroline (0.0353 g, 0.112 mmol), and stirred for a few minutes. Colorless single crystals were obtained after the filtrate had been allowed to stand at room temperature for about two week.

### Refinement

The position of the H atom of the hydroxyl group was located in a difference Fourier map and other H atoms were placed in calculated positions. All H atoms were refined as riding with O—H = 0.87 Å, *U*_iso_ = 1.5*U*_eq_(O) for hydroxyl H, C—H = 0.96 Å, *U*_iso_ = 1.5*U*_eq_(C) for methyl H, and C—H = 0.93 Å, *U*_iso_ = 1.2*U*_eq_(C) for other H atoms.

### Crystal data

**Table:**

[Cd(NO_2_)(C_22_H_20_N_6_)(CH_4_O)]ClO_4_	*Z* = 2
*M**_r_* = 658.34	*F*(000) = 664
Triclinic, *P*1	*D*_x_ = 1.686 Mg m^−^^3^
Hall symbol: -P 1	Mo *K*α radiation, λ = 0.71073 Å
*a* = 8.0241 (17) Å	Cell parameters from 2645 reflections
*b* = 11.580 (2) Å	θ = 2.7–26.3°
*c* = 15.842 (3) Å	µ = 1.00 mm^−^^1^
α = 68.595 (2)°	*T* = 298 K
β = 75.578 (2)°	Prism, colorless
γ = 73.616 (3)°	0.32 × 0.08 × 0.04 mm
*V* = 1297.1 (5) Å^3^	

### Data collection

**Table:**

Bruker SMART APEX CCD diffractometer	4712 independent reflections
Radiation source: fine-focus sealed tube	3966 reflections with *I* > 2σ(*I*)
graphite	*R*_int_ = 0.025
φ and ω scans	θ_max_ = 25.5°, θ_min_ = 1.4°
Absorption correction: multi-scan (*SADABS*; Sheldrick, 1996)	*h* = −9→9
*T*_min_ = 0.740, *T*_max_ = 0.961	*k* = −14→12
6780 measured reflections	*l* = −17→19

### Refinement

**Table:**

Refinement on *F*^2^	Primary atom site location: structure-invariant direct methods
Least-squares matrix: full	Secondary atom site location: difference Fourier map
*R*[*F*^2^ > 2σ(*F*^2^)] = 0.044	Hydrogen site location: inferred from neighbouring sites
*wR*(*F*^2^) = 0.111	H-atom parameters constrained
*S* = 1.02	*w* = 1/[σ^2^(*F*_o_^2^) + (0.0606*P*)^2^] where *P* = (*F*_o_^2^ + 2*F*_c_^2^)/3
4712 reflections	(Δ/σ)_max_ = 0.006
357 parameters	Δρ_max_ = 0.88 e Å^−^^3^
1 restraint	Δρ_min_ = −0.51 e Å^−^^3^

### Special details

**Table:**

Geometry. All e.s.d.'s (except the e.s.d. in the dihedral angle between two l.s. planes) are estimated using the full covariance matrix. The cell e.s.d.'s are taken into account individually in the estimation of e.s.d.'s in distances, angles and torsion angles; correlations between e.s.d.'s in cell parameters are only used when they are defined by crystal symmetry. An approximate (isotropic) treatment of cell e.s.d.'s is used for estimating e.s.d.'s involving l.s. planes.
Refinement. Refinement of *F*^2^ against ALL reflections. The weighted *R*-factor *wR* and goodness of fit *S* are based on *F*^2^, conventional *R*-factors *R* are based on *F*, with *F* set to zero for negative *F*^2^. The threshold expression of *F*^2^ > σ(*F*^2^) is used only for calculating *R*-factors(gt) *etc*. and is not relevant to the choice of reflections for refinement. *R*-factors based on *F*^2^ are statistically about twice as large as those based on *F*, and *R*- factors based on ALL data will be even larger.

### Fractional atomic coordinates and isotropic or equivalent isotropic displacement parameters (Å^2^)

**Table:**

	*x*	*y*	*z*	*U*_iso_*/*U*_eq_	
C1	0.4749 (8)	1.0095 (5)	0.6046 (3)	0.0670 (16)	
H1A	0.3887	1.0840	0.5807	0.101*	
H1B	0.4778	0.9457	0.5787	0.101*	
H1C	0.5887	1.0305	0.5887	0.101*	
C2	0.4278 (6)	0.9603 (4)	0.7065 (3)	0.0425 (11)	
C3	0.2868 (6)	1.0116 (4)	0.7631 (3)	0.0397 (10)	
H3	0.1989	1.0830	0.7445	0.048*	
C4	0.3026 (5)	0.9373 (4)	0.8502 (3)	0.0346 (9)	
C5	0.1895 (7)	0.9562 (4)	0.9363 (3)	0.0490 (12)	
H5A	0.2561	0.9772	0.9692	0.073*	
H5B	0.1501	0.8795	0.9740	0.073*	
H5C	0.0894	1.0240	0.9212	0.073*	
C6	0.5272 (5)	0.7363 (4)	0.9123 (3)	0.0314 (9)	
C7	0.4706 (6)	0.7157 (4)	1.0073 (3)	0.0388 (10)	
H7	0.3818	0.7749	1.0286	0.047*	
C8	0.5485 (6)	0.6075 (4)	1.0668 (3)	0.0386 (10)	
H8	0.5104	0.5910	1.1297	0.046*	
C9	0.7363 (5)	0.5504 (4)	0.9394 (3)	0.0322 (9)	
C10	0.6865 (6)	0.5200 (4)	1.0343 (3)	0.0358 (10)	
C11	0.8843 (6)	0.4699 (4)	0.9010 (3)	0.0353 (9)	
C12	0.7791 (6)	0.4061 (4)	1.0927 (3)	0.0432 (11)	
H12	0.7435	0.3841	1.1560	0.052*	
C13	0.9732 (6)	0.3602 (4)	0.9608 (3)	0.0386 (10)	
C14	0.9175 (6)	0.3300 (4)	1.0570 (3)	0.0457 (12)	
H14	0.9770	0.2570	1.0962	0.055*	
C15	1.1213 (6)	0.2898 (4)	0.9176 (4)	0.0488 (12)	
H15	1.1866	0.2163	0.9535	0.059*	
C16	1.1701 (6)	0.3275 (4)	0.8248 (4)	0.0497 (12)	
H16	1.2676	0.2804	0.7970	0.060*	
C17	1.0714 (6)	0.4380 (4)	0.7719 (3)	0.0410 (11)	
C18	1.3989 (8)	0.3383 (5)	0.6335 (4)	0.0791 (19)	
H18A	1.4800	0.3356	0.5780	0.119*	
H18B	1.3571	0.2607	0.6610	0.119*	
H18C	1.4572	0.3484	0.6756	0.119*	
C19	1.2475 (7)	0.4470 (5)	0.6118 (4)	0.0532 (13)	
C20	1.2191 (7)	0.5289 (5)	0.5292 (4)	0.0585 (15)	
H20	1.2891	0.5264	0.4732	0.070*	
C21	1.0657 (7)	0.6189 (5)	0.5418 (3)	0.0518 (13)	
C22	0.9811 (8)	0.7316 (6)	0.4720 (4)	0.0719 (17)	
H22A	0.8555	0.7452	0.4900	0.108*	
H22B	1.0127	0.7175	0.4135	0.108*	
H22C	1.0204	0.8050	0.4675	0.108*	
C23	1.0818 (8)	0.8475 (7)	0.6558 (5)	0.092 (2)	
H23A	1.1478	0.7659	0.6518	0.138*	
H23B	1.1211	0.8676	0.7003	0.138*	
H23C	1.0994	0.9108	0.5969	0.138*	
Cd1	0.74780 (4)	0.68545 (3)	0.72015 (2)	0.03683 (13)	
Cl1	0.73701 (17)	0.08191 (12)	0.81116 (9)	0.0547 (3)	
N1	0.5259 (5)	0.8567 (3)	0.7550 (2)	0.0380 (8)	
N2	0.4505 (4)	0.8424 (3)	0.8447 (2)	0.0319 (8)	
N3	0.6559 (4)	0.6565 (3)	0.8799 (2)	0.0314 (7)	
N4	0.9316 (5)	0.5065 (3)	0.8091 (2)	0.0370 (8)	
N5	1.1118 (5)	0.4859 (3)	0.6750 (3)	0.0431 (9)	
N6	0.9995 (5)	0.5936 (4)	0.6303 (3)	0.0482 (10)	
N7	0.5172 (6)	0.6276 (5)	0.6397 (3)	0.0642 (13)	
O1	0.8368 (6)	0.1605 (4)	0.8171 (4)	0.0963 (15)	
O2	0.7550 (7)	0.0832 (5)	0.7196 (3)	0.1006 (16)	
O3	0.5589 (6)	0.1235 (5)	0.8444 (4)	0.1071 (17)	
O4	0.7972 (8)	−0.0435 (4)	0.8643 (4)	0.1132 (18)	
O5	0.5944 (5)	0.7160 (4)	0.6005 (2)	0.0651 (10)	
O6	0.5487 (6)	0.5674 (4)	0.7184 (3)	0.0741 (12)	
O7	0.9035 (5)	0.8448 (4)	0.6826 (3)	0.0770 (13)	
H9	0.8513	0.9132	0.6977	0.115*	

### Atomic displacement parameters (Å^2^)

**Table:**

	*U*^11^	*U*^22^	*U*^33^	*U*^12^	*U*^13^	*U*^23^
C1	0.089 (5)	0.059 (3)	0.036 (3)	0.006 (3)	−0.008 (3)	−0.013 (3)
C2	0.050 (3)	0.039 (2)	0.034 (2)	−0.005 (2)	−0.006 (2)	−0.011 (2)
C3	0.033 (2)	0.036 (2)	0.047 (3)	0.0013 (19)	−0.0084 (19)	−0.014 (2)
C4	0.028 (2)	0.033 (2)	0.045 (2)	−0.0069 (18)	−0.0017 (18)	−0.017 (2)
C5	0.049 (3)	0.039 (3)	0.047 (3)	−0.003 (2)	0.007 (2)	−0.015 (2)
C6	0.032 (2)	0.034 (2)	0.033 (2)	−0.0123 (18)	−0.0036 (17)	−0.0124 (18)
C7	0.036 (3)	0.046 (3)	0.035 (2)	−0.008 (2)	−0.0034 (18)	−0.017 (2)
C8	0.042 (3)	0.050 (3)	0.027 (2)	−0.018 (2)	−0.0015 (18)	−0.013 (2)
C9	0.031 (2)	0.033 (2)	0.037 (2)	−0.0123 (18)	−0.0056 (17)	−0.0115 (19)
C10	0.038 (3)	0.038 (2)	0.036 (2)	−0.019 (2)	−0.0091 (18)	−0.0070 (19)
C11	0.032 (2)	0.034 (2)	0.045 (2)	−0.0085 (18)	−0.0090 (19)	−0.015 (2)
C12	0.050 (3)	0.041 (3)	0.039 (2)	−0.020 (2)	−0.014 (2)	−0.002 (2)
C13	0.036 (3)	0.027 (2)	0.057 (3)	−0.0088 (18)	−0.017 (2)	−0.011 (2)
C14	0.052 (3)	0.035 (2)	0.052 (3)	−0.013 (2)	−0.025 (2)	−0.002 (2)
C15	0.044 (3)	0.032 (2)	0.073 (4)	−0.001 (2)	−0.025 (2)	−0.015 (2)
C16	0.039 (3)	0.040 (3)	0.074 (4)	0.001 (2)	−0.010 (2)	−0.028 (3)
C17	0.035 (3)	0.034 (2)	0.060 (3)	−0.0072 (19)	−0.007 (2)	−0.022 (2)
C18	0.061 (4)	0.062 (4)	0.096 (5)	−0.009 (3)	0.030 (3)	−0.036 (3)
C19	0.043 (3)	0.051 (3)	0.072 (4)	−0.020 (2)	0.016 (2)	−0.037 (3)
C20	0.058 (4)	0.064 (3)	0.060 (3)	−0.028 (3)	0.022 (3)	−0.037 (3)
C21	0.053 (3)	0.057 (3)	0.047 (3)	−0.021 (3)	0.007 (2)	−0.021 (3)
C22	0.081 (5)	0.076 (4)	0.049 (3)	−0.023 (3)	0.006 (3)	−0.015 (3)
C23	0.057 (4)	0.104 (5)	0.134 (6)	−0.033 (4)	0.002 (4)	−0.058 (5)
Cd1	0.0373 (2)	0.0375 (2)	0.03387 (19)	−0.00520 (13)	−0.00153 (13)	−0.01417 (14)
Cl1	0.0500 (8)	0.0585 (8)	0.0617 (8)	0.0005 (6)	−0.0168 (6)	−0.0302 (7)
N1	0.039 (2)	0.040 (2)	0.0287 (18)	−0.0008 (16)	−0.0027 (15)	−0.0106 (16)
N2	0.033 (2)	0.0301 (18)	0.0301 (18)	−0.0057 (15)	−0.0016 (14)	−0.0102 (15)
N3	0.0303 (19)	0.0292 (18)	0.0362 (18)	−0.0068 (15)	−0.0036 (14)	−0.0128 (15)
N4	0.034 (2)	0.0342 (19)	0.044 (2)	−0.0069 (16)	−0.0034 (16)	−0.0162 (17)
N5	0.039 (2)	0.040 (2)	0.052 (2)	−0.0075 (17)	0.0034 (18)	−0.0238 (19)
N6	0.049 (3)	0.049 (2)	0.044 (2)	−0.007 (2)	0.0004 (18)	−0.020 (2)
N7	0.066 (3)	0.085 (4)	0.055 (3)	−0.027 (3)	−0.008 (2)	−0.032 (3)
O1	0.082 (3)	0.093 (3)	0.142 (4)	−0.022 (3)	−0.035 (3)	−0.056 (3)
O2	0.142 (5)	0.103 (4)	0.068 (3)	−0.031 (3)	−0.011 (3)	−0.040 (3)
O3	0.048 (3)	0.138 (4)	0.155 (5)	0.006 (3)	−0.012 (3)	−0.091 (4)
O4	0.141 (5)	0.061 (3)	0.127 (4)	0.006 (3)	−0.062 (4)	−0.012 (3)
O5	0.078 (3)	0.068 (2)	0.047 (2)	−0.016 (2)	−0.0138 (19)	−0.0128 (19)
O6	0.094 (3)	0.086 (3)	0.053 (2)	−0.049 (3)	−0.010 (2)	−0.013 (2)
O7	0.056 (3)	0.068 (3)	0.121 (3)	−0.027 (2)	0.023 (2)	−0.060 (3)

### Geometric parameters (Å, °)

**Table:**

C1—C2	1.490 (6)	C17—N4	1.320 (5)
C1—H1A	0.9600	C17—N5	1.415 (6)
C1—H1B	0.9600	C18—C19	1.486 (8)
C1—H1C	0.9600	C18—H18A	0.9600
C2—N1	1.320 (6)	C18—H18B	0.9600
C2—C3	1.393 (6)	C18—H18C	0.9600
C3—C4	1.348 (6)	C19—C20	1.336 (8)
C3—H3	0.9300	C19—N5	1.379 (6)
C4—N2	1.381 (5)	C20—C21	1.395 (7)
C4—C5	1.491 (6)	C20—H20	0.9300
C5—H5A	0.9600	C21—N6	1.323 (6)
C5—H5B	0.9600	C21—C22	1.485 (8)
C5—H5C	0.9600	C22—H22A	0.9600
C6—N3	1.315 (5)	C22—H22B	0.9600
C6—N2	1.406 (5)	C22—H22C	0.9600
C6—C7	1.409 (6)	C23—O7	1.393 (7)
C7—C8	1.356 (6)	C23—H23A	0.9600
C7—H7	0.9300	C23—H23B	0.9600
C8—C10	1.408 (6)	C23—H23C	0.9600
C8—H8	0.9300	Cd1—O7	2.334 (3)
C9—N3	1.350 (5)	Cd1—N4	2.374 (4)
C9—C10	1.393 (6)	Cd1—N3	2.377 (3)
C9—C11	1.442 (6)	Cd1—O5	2.379 (4)
C10—C12	1.430 (6)	Cd1—N6	2.387 (4)
C11—N4	1.345 (5)	Cd1—O6	2.389 (4)
C11—C13	1.402 (6)	Cd1—N1	2.393 (3)
C12—C14	1.352 (7)	Cl1—O4	1.402 (4)
C12—H12	0.9300	Cl1—O3	1.405 (5)
C13—C15	1.415 (7)	Cl1—O1	1.410 (4)
C13—C14	1.416 (6)	Cl1—O2	1.416 (4)
C14—H14	0.9300	N1—N2	1.368 (4)
C15—C16	1.360 (7)	N5—N6	1.382 (5)
C15—H15	0.9300	N7—O5	1.233 (5)
C16—C17	1.394 (6)	N7—O6	1.236 (5)
C16—H16	0.9300	O7—H9	0.8724
			
C2—C1—H1A	109.5	C21—C20—H20	126.0
C2—C1—H1B	109.5	N6—C21—C20	110.0 (5)
H1A—C1—H1B	109.5	N6—C21—C22	121.1 (5)
C2—C1—H1C	109.5	C20—C21—C22	128.9 (5)
H1A—C1—H1C	109.5	C21—C22—H22A	109.5
H1B—C1—H1C	109.5	C21—C22—H22B	109.5
N1—C2—C3	111.3 (4)	H22A—C22—H22B	109.5
N1—C2—C1	120.7 (4)	C21—C22—H22C	109.5
C3—C2—C1	128.1 (4)	H22A—C22—H22C	109.5
C4—C3—C2	106.5 (4)	H22B—C22—H22C	109.5
C4—C3—H3	126.7	O7—C23—H23A	109.5
C2—C3—H3	126.7	O7—C23—H23B	109.5
C3—C4—N2	106.5 (4)	H23A—C23—H23B	109.5
C3—C4—C5	127.3 (4)	O7—C23—H23C	109.5
N2—C4—C5	126.1 (4)	H23A—C23—H23C	109.5
C4—C5—H5A	109.5	H23B—C23—H23C	109.5
C4—C5—H5B	109.5	O7—Cd1—N4	102.12 (14)
H5A—C5—H5B	109.5	O7—Cd1—N3	99.06 (13)
C4—C5—H5C	109.5	N4—Cd1—N3	68.71 (11)
H5A—C5—H5C	109.5	O7—Cd1—O5	111.48 (15)
H5B—C5—H5C	109.5	N4—Cd1—O5	133.19 (12)
N3—C6—N2	114.6 (3)	N3—Cd1—O5	132.70 (13)
N3—C6—C7	122.3 (4)	O7—Cd1—N6	83.66 (13)
N2—C6—C7	123.1 (4)	N4—Cd1—N6	66.38 (13)
C8—C7—C6	118.4 (4)	N3—Cd1—N6	134.51 (13)
C8—C7—H7	120.8	O5—Cd1—N6	85.54 (14)
C6—C7—H7	120.8	O7—Cd1—O6	162.24 (16)
C7—C8—C10	120.7 (4)	N4—Cd1—O6	94.55 (14)
C7—C8—H8	119.7	N3—Cd1—O6	92.62 (12)
C10—C8—H8	119.7	O5—Cd1—O6	51.28 (13)
N3—C9—C10	122.8 (4)	N6—Cd1—O6	97.66 (14)
N3—C9—C11	117.2 (4)	O7—Cd1—N1	77.01 (13)
C10—C9—C11	119.9 (4)	N4—Cd1—N1	133.55 (11)
C9—C10—C8	116.6 (4)	N3—Cd1—N1	65.71 (11)
C9—C10—C12	119.4 (4)	O5—Cd1—N1	86.45 (13)
C8—C10—C12	124.0 (4)	N6—Cd1—N1	154.67 (13)
N4—C11—C13	123.7 (4)	O6—Cd1—N1	95.94 (14)
N4—C11—C9	117.5 (4)	O4—Cl1—O3	109.7 (4)
C13—C11—C9	118.8 (4)	O4—Cl1—O1	109.2 (3)
C14—C12—C10	120.9 (4)	O3—Cl1—O1	109.2 (3)
C14—C12—H12	119.6	O4—Cl1—O2	107.7 (3)
C10—C12—H12	119.6	O3—Cl1—O2	109.6 (3)
C11—C13—C15	115.0 (4)	O1—Cl1—O2	111.5 (3)
C11—C13—C14	120.1 (4)	C2—N1—N2	105.3 (3)
C15—C13—C14	124.8 (4)	C2—N1—Cd1	135.0 (3)
C12—C14—C13	120.8 (4)	N2—N1—Cd1	118.4 (2)
C12—C14—H14	119.6	N1—N2—C4	110.4 (3)
C13—C14—H14	119.6	N1—N2—C6	117.5 (3)
C16—C15—C13	121.3 (4)	C4—N2—C6	132.0 (3)
C16—C15—H15	119.4	C6—N3—C9	119.1 (3)
C13—C15—H15	119.4	C6—N3—Cd1	123.0 (3)
C15—C16—C17	118.8 (5)	C9—N3—Cd1	117.8 (3)
C15—C16—H16	120.6	C17—N4—C11	119.1 (4)
C17—C16—H16	120.6	C17—N4—Cd1	122.8 (3)
N4—C17—C16	122.2 (4)	C11—N4—Cd1	118.0 (3)
N4—C17—N5	114.7 (4)	C19—N5—N6	109.7 (4)
C16—C17—N5	123.2 (4)	C19—N5—C17	132.7 (4)
C19—C18—H18A	109.5	N6—N5—C17	117.6 (3)
C19—C18—H18B	109.5	C21—N6—N5	105.9 (4)
H18A—C18—H18B	109.5	C21—N6—Cd1	135.8 (4)
C19—C18—H18C	109.5	N5—N6—Cd1	118.4 (3)
H18A—C18—H18C	109.5	O5—N7—O6	113.4 (4)
H18B—C18—H18C	109.5	N7—O5—Cd1	97.9 (3)
C20—C19—N5	106.6 (5)	N7—O6—Cd1	97.4 (3)
C20—C19—C18	127.6 (5)	C23—O7—Cd1	133.0 (4)
N5—C19—C18	125.7 (5)	C23—O7—H9	106.8
C19—C20—C21	107.9 (4)	Cd1—O7—H9	118.9
C19—C20—H20	126.0		

### Hydrogen-bond geometry (Å, °)

**Table:**

*D*—H···*A*	*D*—H	H···*A*	*D*···*A*	*D*—H···*A*
O7—H9···O2^i^	0.87	2.02	2.892 (7)	172
C8—H8···O6^ii^	0.93	2.47	3.291 (6)	148

Symmetry codes: (i) *x*, *y*+1, *z*; (ii) −*x*+1, −*y*+1, −*z*+2.
